# An Integrated Analysis of the Rice Transcriptome and Metabolome Reveals Differential Regulation of Carbon and Nitrogen Metabolism in Response to Nitrogen Availability

**DOI:** 10.3390/ijms20092349

**Published:** 2019-05-11

**Authors:** Wei Xin, Lina Zhang, Wenzhong Zhang, Jiping Gao, Jun Yi, Xiaoxi Zhen, Ziang Li, Ying Zhao, Chengcheng Peng, Chen Zhao

**Affiliations:** 1 Rice Research Institute of Shenyang Agricultural University, Key Laboratory of Northern Japonica Rice Genetics and Breeding, Ministry of Education and Liaoning Province, Key Laboratory of Northeast Rice Biology and Genetics and Breeding, Ministry of Agriculture, Shenyang 110866, China; xwRicePh_D@163.com (W.X.); yijun89@syau.edu.cn (J.Y.); xiaoxizhen1991@163.com (X.Z.); 18642074563@163.com (Z.L.); 15909819597@163.com (Y.Z.); 13936809127@163.com (C.P.); buer0524@163.com (C.Z.); 2 Graduate School of Agricultural Science, Tohoku University, Sendai 981-8555, Japan; zhanglina921210@gmail.com

**Keywords:** rice, transcriptome, metabolome, carbon metabolism, nitrogen metabolism, nitrogen use efficiency (NUE)

## Abstract

Nitrogen (N) is an extremely important macronutrient for plant growth and development. It is the main limiting factor in most agricultural production. However, it is well known that the nitrogen use efficiency (NUE) of rice gradually decreases with the increase of the nitrogen application rate. In order to clarify the underlying metabolic and molecular mechanisms of this phenomenon, we performed an integrated analysis of the rice transcriptome and metabolome. Both differentially expressed genes (DEGs) and metabolite Kyoto Encyclopedia of Genes and Genomes (KEGG) pathway analysis indicated that carbon and nitrogen metabolism is significantly affected by nitrogen availability. Further analysis of carbon and nitrogen metabolism changes in rice under different nitrogen availability showed that high N inhibits nitrogen assimilation and aromatic metabolism pathways by regulating carbon metabolism pathways such as the tricarboxylic acid (TCA) cycle and the pentose phosphate pathway (PPP). Under low nitrogen, the TCA cycle is promoted to produce more energy and α-ketoglutarate, thereby enhancing nitrogen transport and assimilation. PPP is also inhibited by low N, which may be consistent with the lower NADPH demand under low nitrogen. Additionally, we performed a co-expression network analysis of genes and metabolites related to carbon and nitrogen metabolism. In total, 15 genes were identified as hub genes. In summary, this study reveals the influence of nitrogen levels on the regulation mechanisms for carbon and nitrogen metabolism in rice and provides new insights into coordinating carbon and nitrogen metabolism and improving nitrogen use efficiency in rice.

## 1. Introduction

Nitrogen is an extremely important and necessary macronutrient for plant growth and development [1]. A large amount of nitrogen fertilizer is used to achieve high productivity in crops, but only 30–40% of the nitrogen fertilizer applied is estimated to be absorbed by plants [2]. Unused compounds released into the environment cause environmental pollution, such as soil acidification, soil hardening, and eutrophication of water [3,4]. Therefore, to sustain high yield and reduce pollution, it is important to improve the nitrogen use efficiency (NUE).

Carbon and nitrogen metabolism are the two most basic metabolic pathways of plants [5]. The dynamic changes of carbon and nitrogen metabolism in plants directly affect the formation and transformation of photosynthetic products, as well as the absorption of mineral nutrients and protein synthesis. Carbon metabolism provides a carbon source and energy for nitrogen metabolism [6], while nitrogen metabolism provides enzymes and photosynthetic pigments for carbon metabolism. Both carbon and nitrogen metabolism require common reducing power, ATP, and a carbon skeleton [7]. Carbon and nitrogen nutrients are absolutely essential for plant growth and development. Comprehensively revealing the genetic and metabolic basis of carbon–nitrogen interactions is important for increasing crop yield and adapting to nutrient stress.

Previous studies have shown that plant carbon and nitrogen metabolism must be closely coordinated [6,8,9,10]. The carbon metabolism of gene expression is closely related to the level of nitrogen supply [11,12,13]. Nitrogen availability is a key determinant for distributing assimilated carbon among synthesis of organic acids, starch, and sucrose [14]. Double labeling (13C/15N) and nuclear magnetic resonance (NMR) analyses have shown that the α-ketoglutarate (2OG), used for GS/GOGAT during the day, derives from stored organic acids (probably malate or citrate) produced during the night, and therefore carbon metabolism is important for nitrogen assimilation [15]. ABC1/OsFd-GOGAT plays a vital role in the growth and development of rice via regulating nitrogen assimilation and the carbon–nitrogen (C/N) balance [16]. Nitrogen use efficiency (NUE) refers to the dry matter produced by plants that absorbs per unit mass of nitrogen and results from the coordination of plant carbon and nitrogen metabolism during development. Maintaining the coordination of carbon and nitrogen metabolism, and an appropriate balance of carbohydrates to nitrogen metabolites, also referred to as the “C/N balance,” is important for plant growth, development, and yield production [6,8,17,18,19]. Therefore, reasonable regulation of carbon and nitrogen metabolism to improve NUE is an effective way to increase rice yield and reduce environmental costs [20,21].

The recent development and application of high-throughput sequencing technologies, high-resolution mass spectrometry technologies, and information processing technologies has led systems biology (omics) research to become predominant in exploring major biological phenomena. In particular, transcript and metabolite datasets have been combined through correlation and clustering analyses and further represented as connection networks between genes and metabolites in many plants, to reveal the response mechanism of rice to elevated night temperature [22], the regulation mechanism of delphinidin in flower color in grape hyacinth [23], the potato pigmentation mechanism [24], the blue flower formation mechanism in waterlily [25], and catechin production in albino tea cultivar “YuJin-Xiang” [26]. However, almost no research has been conducted on the response of rice to nitrogen nutrient supply by integrated analysis of the transcriptome and metabolome. In this study, rice was exposed to low N, control N, and high N for 30 days. We determined the morphological and physiological characteristics, as well as changes in transcription and metabolism among these treatments. The integrated analysis of the rice transcriptome and metabolome allowed us to get more insight into the carbon and nitrogen metabolism regulation mechanism in response to nitrogen availability, which could be of great practical significance in coordinating rice carbon and nitrogen metabolism and increasing NUE.

## 2. Results

### 2.1. Nitrogen Availability Affects the Morphology, Physiology, and Growth Characteristics of Rice Leaves

Compared with control nitrogen, nitrogen deficiency had a negative effect on leaf biomass and leaf area, while high N promoted leaf biomass and leaf area. Nitrogen concentration had no significant effect on Ci. Compared with control nitrogen, low N decreased Chl a, Chl b, Pn, and gs, while high N increased Chl a, Chl b, and gs, but had no significant effect on Pn. The availability of N also affected the nutritional status of the rice leaf. There was a negative effect on free amino content under low N and high N. Compared with control nitrogen, N deficiency led to great decreases in the content of nitrogen and carbon, but had no significant effect on the content of soluble sugar and total protein, whereas high N increased nitrogen, soluble sugar, and total protein content, but had no significant effect on carbon content. Low N increased C/N of the rice leaf compared to control N, whereas high N decreased C/N of the rice leaf as compared to control nitrogen; NUE and PNUE decreased with an increasing nitrogen concentration (Table 1).

### 2.2. Metabolite Profiles of Rice Leaves in Response to Nitrogen Availability

**Figure 1:**
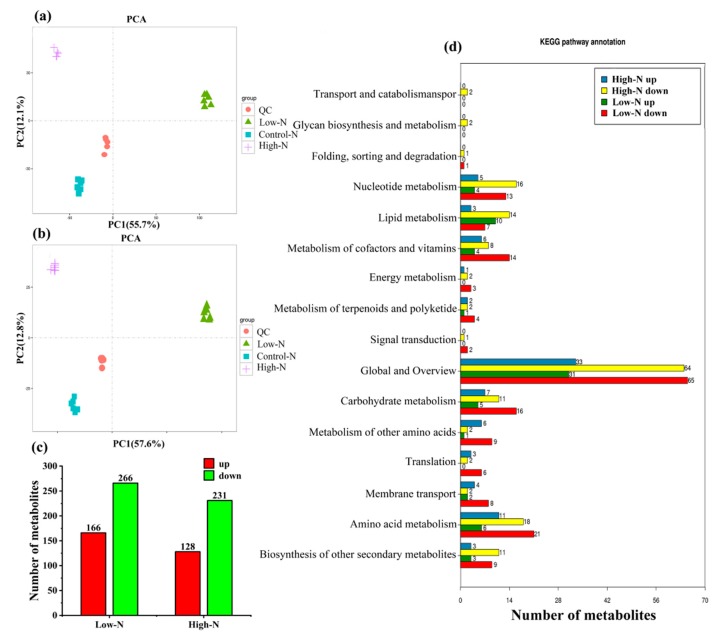
Metabolic analysis of rice leaves under low N and high N: Test samples and quality control samples principal component analysis in (**a**) positive and (**b**) negative ion mode; (**c**) the total number of different metabolites, upregulated and downregulated, under low N and high N; (**d**) Kyoto Encyclopedia of Genes and Genomes (KEGG) analysis of the differentially changed metabolites.

In order to obtain an overview of metabolic changes in response to N, non-targeted metabolic analysis was conducted with LC-ESI-MS/MS. Principal component analysis (PCA) was performed on all samples and QC samples (prepared by equal mixing of all experimental sample extracts) (Figure 1a,b). The dispersion between QC samples showed that the metabolic analysis instrument had stable and reliable data detection and could thus be used for subsequent analysis. To identify metabolites affected by high and low nitrogen, partial least squares discriminant analysis (PLS-DA) and orthogonal partial least squares discriminant analysis (OPLS-DA) were performed on the metabolic profiles (Figure S1), and significantly differential metabolites were selected with variable importance for projection (VIP) > 1. According to the above criterion, compared with the control nitrogen, a total of 557 metabolites were detected under high and low nitrogen. As shown in Figure 1c, compared to the control treatment, 432 metabolites were differentially accumulated under low N treatment; 166 had increased levels and 266 had decreased levels. In total, 359 metabolites were differentially changed under high N treatment; 128 had increased levels and 231 had decreased levels. The decreased levels of metabolites were higher than the increased levels of metabolites under both high and low nitrogen.

To further understand the functions of the differentially changed metabolites and the related biological processes they participate in, pathway enrichment analysis of the differentially changed metabolites was conducted using KEGG. Significantly enriched metabolites were identified in “Global and Overview,” “Amino acid metabolism,” and “Carbohydrate metabolism” (Figure 1d). This result indicated that the carbon and nitrogen metabolism of rice leaves was significantly affected by nitrogen nutrient supply. To further demonstrate how carbon and nitrogen metabolism respond to nitrogen nutrition, 33 metabolites involved in nitrogen metabolism, amino acid metabolism, and carbohydrate metabolism (starch and sucrose metabolism, citrate cycle, glycolysis, pentose phosphate pathway, and carbon fixation in photosynthetic organisms) were identified based on the retention time (rT) and molecular weight in the extracted ion chromatogram (EIC).

### 2.3. Transcriptome Profiles of Rice Leaves in Response to Nitrogen Availability

**Figure 2:**
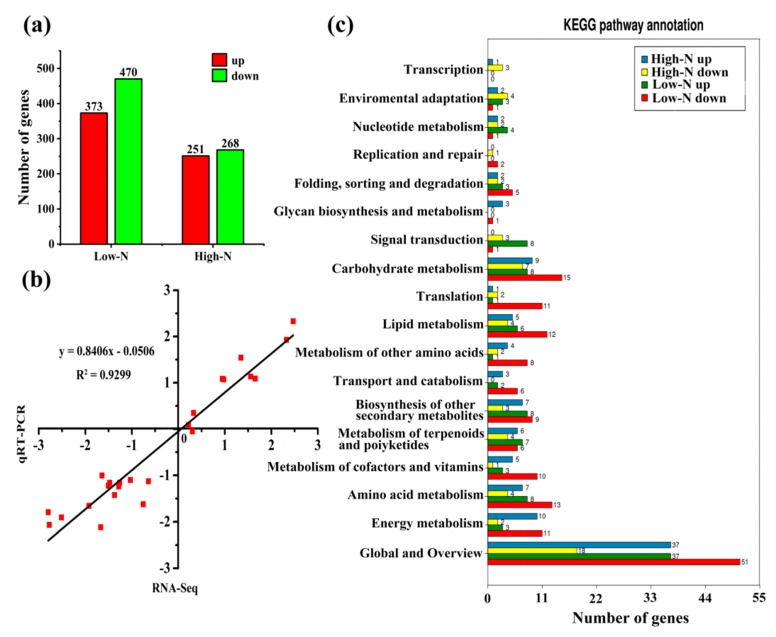
Transcriptional analysis of rice leaves under low nitrogen and high nitrogen: (**a**) The total number of differentially expressed genes (DEGs), upregulated and downregulated, under low nitrogen and high nitrogen; (**b**) a qRT-PCR assay was carried out for 12 randomly selected DEGs. Values are the log_2_ (FC) (low N/control N or high N/control N) for genes. The correlation coefficient (*R*^2^) is indicated in the figure; and (**c**) KEGG analysis of DEGs.

The expression profiles rice under low N and high N were analyzed by RNA-seq (Figure 2a). Compared to the control treatment, 843 genes were differentially expressed under low N, with 373 upregulated and 470 downregulated. Under high N, 519 genes were differentially expressed, with 251 upregulated and 268 downregulated. The number of downregulated genes was higher than the number of upregulated genes under both high and low nitrogen. We randomly selected 12 DEGs for qRT-PCR. Correlation analysis showed that the qRT-PCR results were highly correlated with the transcriptome profiles, and the Pearson coefficient was 0.93 (Figure 2b). This result showed that the RNA-Seq data were reliable.

To further understand the functions of the DEGs and the related biological processes they participate in, GO and KEGG enrichment analyses were conducted. GO analysis classified DEGs under low N and high N into ‘molecular function,’ ‘cellular component,’ and “biological process”, involving 33 GO terms (Figure S2). Within molecular function, the enriched GO terms were “catalytic activity” and “binding”. Within cellular component, the enriched GO terms were “cell”, “cell part”, and “organelle”. Within biological process, the enriched GO terms were “metabolic process”, “single-organism process”, and “cellular process”. Pathway enrichment analysis of the DEGs identified in the present study using KEGG identified significantly enriched “amino acid metabolism”, “biosynthesis of secondary metabolites”, “carbohydrate metabolism”, “energy metabolism”, and “lipid metabolism” by comparing these genes with the whole genomic background (Figure 2c). The transcription and metabolism analysis results consistently showed that N availability significantly affected rice amino acid metabolism and carbohydrate metabolism.

### 2.4. Nitrogen Assimilation and Amino Acid Metabolism

**Figure 3:**
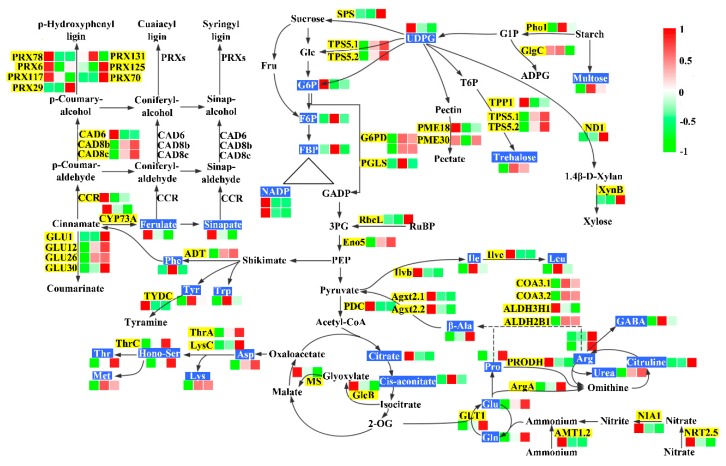
Carbon and nitrogen metabolism pathways overrepresented among differentially expressed genes and significantly changed metabolites. Black characters with yellow background are genes, while white characters with blue background are metabolites. The three squares under the gene and metabolites names indicate expression abundance or metabolite levels of low N, control N, and high N.

Nitrogen availability affected the internal nitrogen compounds of rice leaves, such as the concentrations of total protein and total free amino acids (Table 1). Consistently, the integrated analysis of transcriptome and metabolome showed that, compared with normal nitrogen, nitrogen transport, assimilation, and amino acid metabolism changed significantly under low and high nitrogen (Figure 3). Ammonium transporter gene *OsAMT1.2* and nitrate transporter gene *OsNRT2.5* were upregulated under low N, while nitrate transporter gene *OsNRT2.5* was downregulated under high N. The gene encoding nitrate reductase (*OsNIA1*) was induced by low N but inhibited by high N. The gene encoding NADH-dependent glutamate synthase (*OsGLT1*) was down regulated under low N but upregulated under high N. The metabolome profile showed that glutamine and glutamate were decreased under low N and increased under high N.

We found that the free amino acids of rice decreased under high and low nitrogen (Table 1). The analysis of metabolic profile results (Figure 3) showed that levels of three aromatic amino acids (Tyr, Trp, Phe), five glutamate family amino acids (Glu, Gln, l-Arg, d-Arg, Pro), two branched-chain amino acids (Lue, Ile), five aspartate family amino acids (Asp, Lys, Thr, Met, D1-homoserine), and three nitrogenous metabolites (β-ala, citrulline, GABA) were reduced under low N. The levels of three aromatic amino acids (Tyr, Trp, Phe) and two branched chain amino acids (Lue, Ile) decreased under high N, whereas five glutamate family amino acids (Glu, Gln, l-Arg, d-Arg, Pro), three aspartate family amino acids (Asp, Thr, D1-Homoserine), and three nitrogenous metabolites (β-ala, citrulline, urea) had increased accumulation under high N. d-Methionine and GABA had decreased accumulation under high N. Consistent with metabolome profiles, N deprivation decreased the transcript levels of the genes *OsGLT1*, *OsArgA*, *OsCOA3.1*, *OsCOA3.2*, *OsALDH2b1*, *OsCAD*, *OsThrA*, *OsThrC*, and *OsLysC*, and increased the transcript levels of *OsTYDC*, *Agxt2.1*, *Agxt2.2*, *OsIlvb*, and *OsIlve*. This result indicated that low N inhibited the amino acid synthesis process and promoted the amino acid degradation process. Excess N increased the transcript levels of *OsGLT1*, *OsArgA*, *OsLysC*, and *OsSAT* and decreased the transcript levels of *OsALDH3H1*. Cinnamyl alcohol dehydrogenase, trans-cinnamic acid 4-monooxygenase, and cinnamoyl-CoA reductase are key enzymes in phenylpropanoid biosynthesis. *OsCAD8B*, *OsCAD8C*, *OsCYP73A*, and *OsCCR* expression were induced by low N, and *OsCYP73A* expression was inhibited by high N. In addition, the contents of ferulate and sinapate increased under low N and the content of ferulate decreased under high N.

### 2.5. Carbon Metabolism

**Figure 4:**
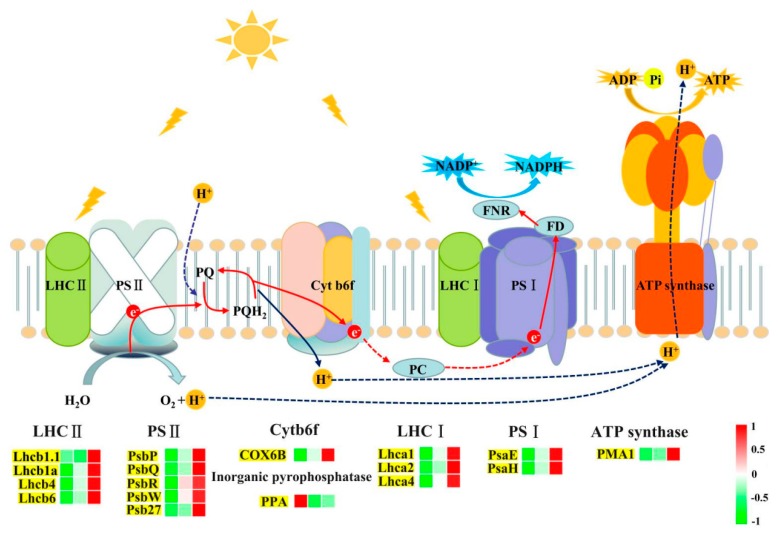
Expression patterns of photosynthetically related genes under low nitrogen and high nitrogen. The three squares under the gene names indicate expression abundance of low N, control N, and high N. Image of photosynthetic electron transport originated from *Plant Physiology*, 5th edition [27].

As shown in Figure 4, the photosynthetic electron transport genes *OsLhca1*, *OsLhca4*, *OsLhcb1*, *OsLhcb4*, *OsPsaE*, *OsPsaH*, *OsPsbQ*, *OsPsbR*, *OsPsbW*, and *OsCOX6B* were downregulated under low N. Expression of the inorganic pyrophosphatase (*OsPPA*) encoding gene was upregulated under low N, whereas, *OsLhca2*, *OsLhcb1*, *OsLhcb6*, *OsPsbP*, *OsPsbQ*, and *OsPsb27* for photosynthetic electron transport were upregulated under high N. Further, ATPD of ATP synthase (*OsPMA1*) and ribulose-bisphosphate carboxylase small chain (*OsRbcL*) encoding gene were upregulated by high N. Collectively, our results show relatively lower photosynthetic capacity under low N. Even if the expression of photosynthesis-related genes were higher under high N, the photosynthesis rate had no significant increase, and the PNUE decreased as the nitrogen level increased.

Glycolysis, PPP, and the tricarboxylic acid (TCA) cycle are primary metabolic pathways in plants, supplying energy and carbon skeletons for other metabolic pathways (Figure 3). Six differentially accumulated metabolites and seven differentially expressed genes (DEGs) were identified in the above pathways under high and low nitrogen. In glycolysis, downregulation of *OsEno5* under low N was detected, while *OsPDC* was upregulated. G6P was more abundant under low nitrogen, whereas F6P and FBP were lower under the two treatments. NADP showed increased accumulation under low N. In the PPP, two *OsG6PD* (XLOC_034644, XLOC_034644) genes were down-regulated under low N and *OsPGLS* was downregulated under high and low nitrogen. This finding indicated lower NADPH production through PPP under high and low nitrogen. In the TCA cycle, citrate had increased accumulation under low nitrogen, while cis-Aconitate had decreased accumulation under high and low nitrogen. The key genes of the glyoxylate metabolism pathway *OsMS* and *OsGlcB* were downregulated under high nitrogen. These results indicated that the TCA cycle was promoted to produce more energy under low nitrogen, while high N inhibited the TCA and glyoxylate metabolic pathways.

Starch and sucrose are among the main carbohydrates in rice (Figure 3). We found three differentially accumulated metabolites and 14 DEGs involved in starch and sucrose metabolism. The accumulation of maltose and trehalose were lower and UDP-glucose was higher under low N. Maltose also had reduced accumulation under high N. The trehalose synthesis genes *OsTPS1* and *OsTPS5* were upregulated and the trehalose catabolism gene *OsTPP1* was suppressed under high N. The abundance of the sucrose and xylose synthesis genes *OsSPS*, *OsND1*, and *OsXynB* were enhanced under high N. The pectate synthesis-related genes *OsPME18* and *OsPME30* were upregulated under low N; *OsPME30* was also upregulated under high N. The starch catabolism gene *OsPho1* was inhibited by low N and *OsGlgC* was inhibited by high N. In addition, polysaccharide catabolism-related genes and all four GLU genes were inhibited by low N, and all except *OsGLU26* were promoted by high N.

### 2.6. Regulation of Differential Gene Expression by Transcription Factors

Transcription factors (TFs) can regulate the expression of other genes and play an important role in regulating plant growth and adapting to biotic and abiotic stresses. As shown in Table 2, a total of 65 TFs have been identified in rice leaves exposed to high and low nitrogen, covering 22 families of TFs (Table S2). Overall, most TFs were upregulated under low N and not changed or downregulated under high N. The bHLH, NAC, and WRKY TF families are relatively large TF families responsive to N deficiency. This result indicates that there are some differences in the response of TFs to nitrogen availability. Although the NAC family members were responsive to different nitrogen availability, six NAC TFs were detected in low N, five of which were upregulated, whereas three NAC family TFs detected under high N conditions were downregulated.

### 2.7. Co-Expression Networks Reveal a Differential Regulatory Network of Carbon and Nitrogen Metabolism under Low N and High N

**Figure 5:**
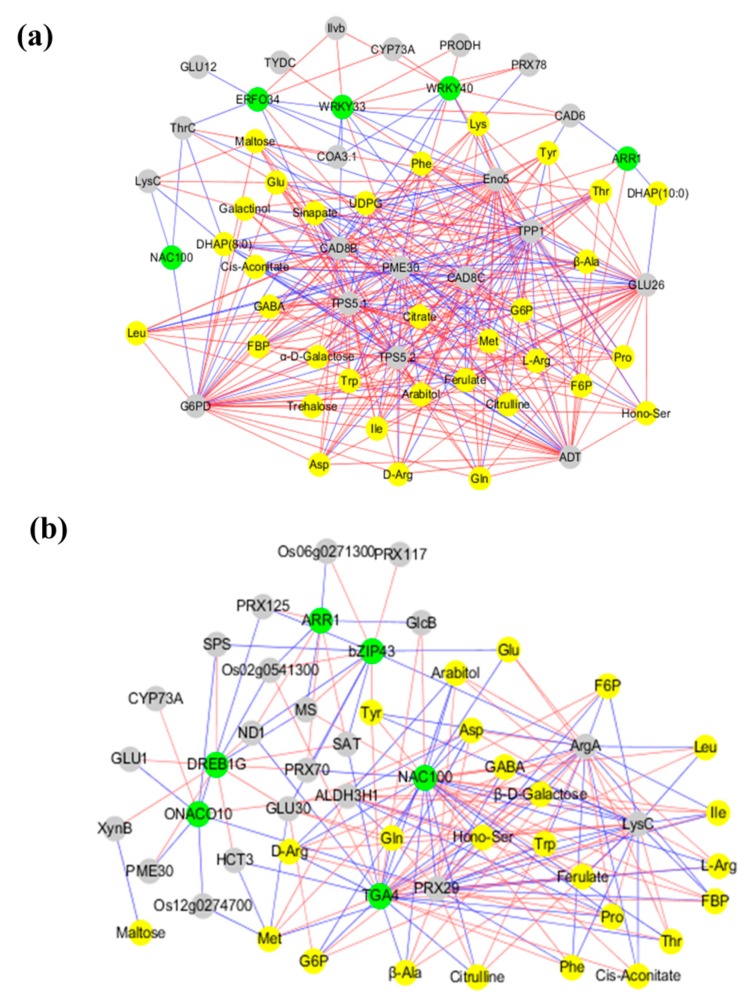
The Pearson correlation network reveals the regulatory mechanisms of carbon and nitrogen metabolism. (**a**) Co-expression network under low nitrogen; (**b**) co-expression network under high nitrogen. Different colors of nodes represent metabolites (yellow), genes (gray), and TFs (green). Red edges represent positive correlations and blue edges represent negative correlations.

To understand the regulatory network of carbon and nitrogen metabolism under low N and high N, we first screened nine TFs based on the targeting relationship between TFs and genes involved in carbon and nitrogen metabolism; of these genes, *OsARR1* and *OsNAC100* were detected under both low N and high N. Then, we selected 35 carbon and nitrogen metabolites and 63 genes (nine TFs screened above; 54 transcripts involved in carbon and nitrogen metabolism) for Pearson correlation analysis. We plotted all pairs of regulatory relationships using a threshold of a Pearson correlation coefficient greater than 0.95 (Figure 5). The visualization in Cytoscape revealed that a total of 59 nodes were connected in the network with 344 edges under low N. A total of 54 nodes were connected in the network with 182 edges under high N. According to the edge greater than 20, we obtained 15 hub genes for regulating carbon and nitrogen metabolism: *OsTPS5.1*, *OsTPS5.2*, *OsTPP1*, *OsG6PD*, *OsEno5*, *OsCAD8C*, *OsADT*, *OsGLU26*, *OsPME30*, and *OsCAD8B* were identified under low N; *OsNAC100*, *OsTGA4*, *OsLysC*, *OsArgA*, and *OsPRX29* were identified under high N.

## 3. Discussion

Previous studies have shown that rice transcriptomic expression levels were affected by variations in nitrogen availability [28,29,30], but these studies focus on the influence of nitrogen deficiency. Unlike these studies that consider low nitrogen treatment, in the current study a high nitrogen treatment was carried out, and the difference changes metabolites were also analyzed with LC-ESI-MS/MS analysis. We observed that rice experiences a large difference in response to low nitrogen and high nitrogen: there are many differences in the morphology, physiology, gene expression, and metabolite changes of rice between low nitrogen and high nitrogen. Clearly, morphological and physiological adaptation to environmental conditions is driven by changes in gene regulation and metabolite accumulation. Despite, the substantial difference in metabolites levels and genes expression between the three environment conditions used in this study, the majority of the observed metabolite changes was in accordance with the transcriptome profile, suggesting that a significant number of the respective metabolic processes might be regulated at the transcriptional level. However, there are exceptions for some reactions such as Ile and Leu, where gene expression and some metabolites levels showed opposite trends. In this case the enzymes encoded by these genes are likely to be controlled by post-transcriptional or past-translational processes [31,32].

Inorganic nitrogen is absorbed and transported by specific transfer proteins, such as ammonium transporters (AMTs) and nitrate transporters (NRTs) [33]. For many plants, only a little nitrate is assimilated in roots, while a larger portion is assimilated in leaves [34]. Nitrate is reduced to nitrite by nitrate reductase (NR) and nitrite is converted to ammonium by nitrite reductase (NIR). The ammonium is incorporated into amino acids, mainly through glutamine synthetase (GS) and glutamate synthase (GOGAT) in plants [35]. Most of the studies have implicated nitrogen transport genes, nitrogen assimilation genes, and GS/GOGAT cycle genes involved in nitrogen use efficiency of rice [4,6,16]. In the current study, we found that nitrogen transport and assimilation were promoted by low nitrogen, while were inhibited by high nitrogen. Interestingly, our results showed that the contents of Glu and Gln were inhibited by low nitrogen and promoted by high nitrogen. Previous studies have also shown that Glu accumulation inhibits the expression of nitrate reduction and transport genes [36,37]. Therefore, our results demonstrate that nitrogen transport and assimilation are adjusted by the GS/GOGAT cycle in response to nitrogen availability.

Compared with control nitrogen supply, we observed that the detected amino acid content decreased under low nitrogen. Interestingly, under high-nitrogen conditions, the content of aromatic amino acids and branched chain amino acids decreased, and amino acids related to major nitrogen metabolism were significantly increased. This indicates that under high-nitrogen conditions, excess assimilated nitrogen did not enter the synthesis of aromatic amino acids through the central carbon metabolic pathway. Instead, high N was redirected to the amino acid metabolism pathway associated with nitrogen metabolism, such as glutamate family amino acids, aspartate family amino acids, and the urea cycle, which, in turn, caused an imbalance of nitrogen in plants. In addition, we found that nitrogen availability significantly affects phenylpropanoid biosynthesis in rice. The phenylpropanoid metabolic pathway is closely related to the synthesis of lignin [38], and the biosynthesis pathway of lignin is closely related to the utilization of photosynthetic carbon [39]. Our results could indicate that under low-nitrogen conditions, photosynthetic carbon can be transferred to lignin synthesis via glycolysis or PPP, while high nitrogen inhibited this process. Therefore, it is possible that nitrogen affects the rice C/N balance by regulating the distribution of photosynthetic carbon and assimilation of nitrogen to aromatic amino acids and phenylpropanoid biosynthesis. In turn, this affects the rice yield and nitrogen use efficiency.

Many studies have demonstrated that there is a good positive correlation between plant NUE and PNUE [39,40,41]. Therefore, understanding the reduction of PNUE under high nitrogen has important significance for the improvement of rice NUE. In the current study, the expression level of photosynthesis-related genes also increased significantly with an increasing nitrogen supply level. Previous studies with different species have found that the transcriptional abundances of some photosynthesis-related genes can be influenced by nitrogen availability [13,42]. Some studies have shown lower photosynthetic gene expression or protein abundance under low-nitrogen conditions, which may occur to avoid energy loss from photosynthetic systems [43,44]. Interestingly, we found that the transcription levels of photosynthesis-related genes are increased under high nitrogen, while PNUE was significantly reduced compared to control nitrogen. Zhang et al.’s [39] study of poplar cell suspension showed that photosynthesis-associated proteins act as nitrogen pools in poplar cells when nitrogen supply is sufficient. Li et al. [45] study of rice found that Rubisco serve as nitrogen pool under high nitrogen additional to its function in photosynthesis. Therefore, we speculate that these genes encode proteins which are either involved in photosynthesis or serve as nitrogen pool. This may be the reason why the PUNE decreases with nitrogen supply.

The pentose phosphate pathway (PPP) is a major pathway that generates reducing power; the main regulatory enzymes of PPP are 6-phosphogluconolactonase (*OsPGLS*) and glucose 6-phosphate dehydrogenase (*OsG6PD*) [2]. Transcription levels of *OsG6PD* and *OsPGLS* indicate that PPP is inhibited by low N and high N. Zhang et al. [39] also found that the absolute flux through the oxidative pentose pathway was substantially lower under low nitrogen. Erythrose-4P is a metabolite of PPP and further converts it into an aromatic amino acid. Therefore, PPP is closely related to the synthesis of aromatic amino acids. In the current study, we also found that the PPP changes were consistent with the content of aromatic amino acids. Meanwhile, PPP can produce a large amount of NADPH, which provides the main reducing power for various synthesis reactions of cells, such as nitrate reduction [39] and antioxidant systems [46]. Our observations have shown that, under high nitrogen, both PPP and nitrate reduction processes were inhibited. Interestingly, despite low nitrogen inhibiting PPP, nitrate assimilation was enhanced. This probably occurred because lower NADPH production by PPP is consistent with the lower reducing power requirements under low nitrogen. In the current study, nitrogen deficiency induced *OsPDC*. Higher expression of *OsPDC* enhances downstream components of the TCA cycle. Assimilation of nitrogen into the amino acid metabolic pathway through the TCA cycle, meanwhile, can provide 2-OG, supplying the GS/GOGAT cycle for ammonium re-assimilation [16,47]. Collectively, these observations indicate that the TCA cycle enhancement enables a more rational allocation of nitrogen for the synthesis of various amino acids and provides more 2-OG enhanced ammonium re-assimilation, to enhance rice adaptation to low-nitrogen stress. The TCA cycle and PPP pathway were inhibited by high nitrogen in the present study, indicating that rice production capacity and carbon skeleton synthesis will be inhibited under long-term high nitrogen stress.

Transcription factors play an important role in plant resistance to biotic and abiotic stresses [48,49,50]. Yang et al. [29] used RNA-Seq analysis and detected 28 TF families, with a total of 85 TFs under nitrogen deficiency that play an important role in nitrogen deficiency response and plant growth. In the current study, TF families such as bHLH, MYB-related, NAC, and WRKY were detected to respond to nitrogen availability, of which the WRKY family specifically responded to nitrogen deficiency. Peng et al. [51] found that nitrogen deficiency promoted the expression of NAC29 in *Arabidopsis*. MYB1 is a R2R3-type MYB TF that enhances NRT, NAR, NIR, and GS expression under low nitrogen stress in *Cyanidioschyzon merolae* [52]. In addition, we found that most TFs were upregulated under low nitrogen and downregulated under high nitrogen. This result showed that there is a significant difference in the transcriptional regulation mechanisms of rice between low-nitrogen and high nitrogen. Co-expression network analysis obtained 15 hub genes, indicating that these genes can regulate carbon and nitrogen metabolism in response to changes in nitrogen availability. In future studies it will be important to determine the role of these genes in low or high nitrogen for improving rice yield and nitrogen use efficiency.

In this study, based on comparisons of rice growth with metabolome and transcriptome profiles across three environmental conditions (N levels), we observed that rice experiences a large difference in response to low nitrogen and high nitrogen, of which the changes in carbon and nitrogen metabolism were the most obvious. Integrative analysis of transcriptomic and metabolomic data indicated that mechanisms for improving NUE may involve a redirection of abundant nitrogen to aromatic metabolic pathways, and extensive downregulation of potentially redundant genes that encode photosynthetic and light-harvesting proteins. Moreover, the co-expression network analysis was used to obtain 15 hub genes. This work not only provides insights into the molecular and biochemical mechanisms of rice’s response to nitrogen availability but may be of significance in uncovering the as yet unknown hub gene for carbon and nitrogen metabolism, benefiting the breeding of high-NUE rice cultivars.

## 4. Materials and Methods

### 4.1. Plant Materials and N Treatment

The experiment was conducted at a farm belonging to Shenyang Agricultural University (41°49’N, 123°34’E). Rice (Japonica cv Shennong265) seeds were sterilized and germinated at 28 °C for 2 d, then further grown in an N-sufficient nutrient solution in a growth chamber (28 °C/25 °C and 10 h light/14 h dark). At the three-leaf heart stage, seedlings with the same growth were selected, supplied with 13.33 ppm (Low N), 40 ppm (Control N), or 120 ppm (High N) of nitrogen using NH_4_NO_3_ as the source, and grown under natural conditions for 30 days during the growing season; each group included 30 seedlings. The hydroponic nutrient solution was formulated using the conventional nutrient solution formulation of the International Rice Research Institute (IRRI) with a slight improvement [45]. The pH of the nutrient solution was maintained between 5.45 and 5.55. The pH was adjusted once a day with 1 M NaOH and 1 M HCl. The nutrient solution was changed every three days. After 30 days, the relevant indicators were determined.

### 4.2. Analysis of Leaf Characteristics

Leaves were harvested after 30 days of treatment. Leaf length and width were measured with a ruler and leaf areas were calculated by length · width · 0.75. Then, the leaves were dried at 80 °C to a constant weight, and leaf biomass was measured with a balance. Samples were then powdered with a micro-pulverizer (FZ102, Tianjin, China) and an element analyzer (Elementar Vario MACRO cube, Hanau, Germany) was used to determine the leaf carbon and nitrogen contents. Nitrogen use efficiency (NUE, g·g−1) was calculated as NUE = dry matter accumulation (g)/nitrogen accumulation (g) [53].

Thirty days after nitrogen treatment started, the photosynthetic rate (Pn), intercellular CO_2_ concentration (Ci), and stomatal conductance (gs) of first leaf after nitrogen treatment were measured from 09:00 to 11:00 using a CIRAS-3 portable photosynthetic instrument (PP Systems, Amesbury, MA, USA). Leaf temperature during measurements was maintained at 27 °C; leaf chamber humidity during measurements was maintained from 48% to 50%, with a photosynthetic photon flux density of 1200 μmol m^−2^·s^−1^.The concentrations of chlorophyll a and b were determined based on Li et al.’s method [45]. Photosynthetic NUE (PNUE, μmol g^−1^·s^−1^) was calculated as PNUE = Photosynthetic rate (μmol m^−2^·s^−1^) / leaf N content (g·m^−2^) [54].

The free amino acid content of leaves was determined as described by Cao et al. [55]. The concentrations of soluble sugar of leaves were determined according to the method of Maness [56]. Total protein was measured with a BCA Protein Assay Kit (Shanghai Enzyme-linked Biotechnology, Shanghai, China) based on the operational guidelines of the manufacturer.

Statistical tests were performed with SPSS 19.0 (Softonic International, Barcelona, Spain) statistical software. The data were tested to confirm their normality before the statistical analyses. For experimental variables, one-way ANOVA was applied to assess differences among treatments. Significant differences (*p* < 0.05) between treatments were indicated by different letters according to the ANOVA *F*-test.

### 4.3. Metabolite Extraction and Liquid Chromatography Electrospray Ionization Tandem Mass Spectrometry (LC-ESI-MS/MS) Analysis

Three samples of rice first leaf under low N, control N, and high N treatment for 30 days were measured; six biological replicates per sample were used for metabolic analyses. The freeze-dried samples were ground using a grinder (JXFSTPRP-24., Jingxin Technology, Shanghai, China) equipped for metabolic analyses. Metabolites were extracted with methanol [57]. A 100-μL sample was taken, 300 μL of methanol and 20 μL of internal standard (2-Chloro-l-phenylalanin) were added, and the mixture was vortexed for 30 s and then ultrasonically extracted in an ice water bath for 5 min. The samples were then left to stand at −20 °C for two hours. After centrifugation at 13,000 rpm for 15 min at 4 °C, 200 μL of the supernatant was taken in a 2-mL vial for LC-ESI-MS/MS analysis.

LC-ESI-MS/MS analysis consisted of an ultrahigh-performance liquid chromatography system (UHPLC Agilent 1290, Santa Clara, CA, USA) fitted with a high-resolution mass spectrometer (Q Exactive Orbitrap, Santa Clara) equipped with an ESI interface. An ACQUITY UPLC HSS T3 column (1.7 μm 2.1*100 mm, Waters, Milford, MA, USA) was used for chromatographic separation. The sample injection volume was 1 μL. Spectra were obtained using positive ion mode (POS) and negative ion mode (NEG). The ESI ion source spray voltage was 3800 V (POS) or −3100 V (NEG). The capillary temperature was maintained at 320 °C. Sheath gas and auxiliary gas flow rates were 45 and 15 Arb, respectively. The scan range was from 70 to 1000 m/z with primary resolution of 70,000 and secondary resolution of 17,500. Data analysis was conducted using ProteoWizard (version 3.0.6839, Palo alto, CA, USA) and XCMS (version 1.22.01, University of California, CA, USA) software. Metabolites were identified based on software self-built databases (OSI-SMMS, version 1.0, Dashuo, Dalian, China) and public databases (HMDB, METLIN, KEGG) [58,59]. We combined the OPLS-DA of the VIP values and the univariate statistical analysis of the *t*-test *p* values to screen for significant differential metabolites between the different comparison groups [60]. VIP ≥ 1 and *t*-test *p* < 0.05 were used as a threshold to identify the significantly different metabolites.

### 4.4. RNA Extraction and Transcriptome Sequencing Analysis

Three samples of rice first leaf under low N, control N, and high N treatment were measured for 30 days; three biological replicates per sample were used for transcriptome analyses. Total RNA was extracted with TRIzol reagent (Thermo Fisher Scientific, Waltham, MA, USA). After total RNA was extracted, mRNA was purified using Oligo(dT) beads, then the purified mRNA was fragmented into short fragments using fragmentation buffer and reverse-transcribed into cDNA by random hexamer primers. Second-strand cDNAs were synthesized using DNA polymerase I, RNase H, dNTP, and a buffer. Then the cDNA fragments were purified using a QiaQuick PCR extraction kit, end repaired, poly(A) was added, and the fragments were ligated to Illumina sequencing adapters. The ligation products were size selected by 1% agarose gel electrophoresis, PCR amplified, and sequenced using Illumina HiSeqTM 2500 (Guangzhou, China). In total, nine samples (three nitrogen treatments × three biological replicates) were sequenced. Reads with connectors, reads with more than 10% unknown nucleotides (N), reads with all A bases, and low-quality reads (number of bases with Q ≤ 20 more than 50%) were removed from the data set. We then compared the reads to the ribosomal RNA database (mismatch number: 0) in Bowtie2 (version 2.2.8, GitHub, San Francisco, CA, USA) [61], removing the reads of the aligned rRNA; the retained reads were used for subsequent analysis. The retained reads were aligned to the rice reference genome (Nipponbare) by Tophat2 program (version 2.0.3.12, University of Maryland, Washington D.C., USA) [61]. The transcripts were assembled using Cufflinks (Berkeley, CA, USA) to obtain known transcripts and new transcripts [62]. Then, differential expression analysis across samples was conducted in the edgeR package (http://www.r-project.org/) to obtain DEGs (FDR < 0.05, |log2FC| > 1) [63]. Gene ontology (GO) and Kyoto Encyclopedia of Genes and Genomes (KEGG) analysis of the DEGs were implemented to identify significantly enriched GO terms and metabolic pathways. GO enrichment analysis was conducted in the GOSeq (Lanzhou, China) R program (http://www.r-project.org/) and KEGG pathway analysis was conducted using the KEGG (Kyoto, Japan) Orthology program (http://kobas.cbi.pku.edu.cn/) [64]. We used the *p*-value calculated by the hypergeometric test and corrected by FDR, taking FDR ≤ 0.05 as a threshold to identify the significant functional categories and metabolic pathways.

### 4.5. Co-Expression Network Analysis of Genes and Metabolites

Using the differentially expressed genes as candidate target gene sets, the transcription factor information of rice was found in the Plant TFDB database, and the differential transcription factor information was found by the gene ID number. The MEME (version 4.12.0, University of Nevada, Reno, NV, USA) software was used to find the conserved domain motif of the transcription factor. Then, according to the FIMO (Find Individual Motif Occurrences) analysis tool in MEME (version 4.12.0, University of Nevada, Reno, NV, USA), the TFBS of the 2 kb region (potential promoter region) upstream of the candidate target gene is searched to determine the sequence targeting relationship between the transcription factor and the target gene. Co-expression network analyses were conducted to determine the relationships among genes [65]. According to the method of Zhang et al. [65], Cytoscape software (version 3.3.0, Cytoscape, San Diego, CA, USA) was used to construct a co-expression regulation network of TFs, genes, and metabolites. The co-expression network map was made with *p* > 0.95 as the threshold [66]. The hub genes within the network were identified according to the topological coefficient of each node with degree >20.

### 4.6. Confirmation of Transcriptome Data Using qRT-PCR Analysis

The cDNA was synthesized with total RNA using SYBR Green qPCR (TAKARA, Tokyo, Japan) according to the manufacturer’s protocol. Real-time PCR was conducted with SYBR Premix Ex Taq II (TAKARA, Tokyo, Japan) in an Applied Biosystems QuantStudio 3 (Thermo Fisher Scientific) using transcript-specific primers (Table S1). Relative quantification analysis was conducted using a relative standard curve for threshold values (CT). qRT-PCR data were standardized with *OsACTIN1* as an internal reference. Mapping and calculating correlation coefficients for RNA-Seq and qRT-PCR data were done using the Origin (version 9, Northampton, MA, USA) mapping software.

## Figures and Tables

**Table 1:** Morphology, physiology, and growth response to low nitrogen and high nitrogen. Values labeled with different letters in same row indicate significant difference between the nitrogen treatments. *p* Values of the ANOVAs are indicated. F*^P^*: F Valve *^P^* ^Value^; ns: No significant; * *p* < 0.05; ** *p* < 0.01; *** *p* < 0.001.

Treatments	Low N	Control N	High N	F*^P^* Value
Leaf biomass (g)	1.26 ± 0.11^b^	1.87 ± 0.08^a^	1.81 ± 0.08^a^	42.22 ***
Leaf area (cm^2^)	238.36 ± 20.17^b^	352.20 ± 15.25^a^	341.51 ± 14.24^a^	53.72 ***
Chlorophyll a (Chl a, mg·g^−1^)	0.82 ± 0.03^c^	1.58 ± 0.08^b^	1.73 ± 0.01^a^	324.32 ***
Chlorophyll b (Chl b, mg·g^−1^)	0.37 ± 0.01^c^	0.71 ± 0.04^b^	0.80 ± 0.01^a^	301.64 ***
Intercellular CO_2_ concentration (Ci, μmol·mol^−1^)	285.33 ± 16.07^a^	292.67 ± 4.51^a^	292.67 ± 2.31^a^	0.57 ^ns^
Photosynthetic rate (Pn, μmol m^−2^·s^−1^)	18.13 ± 0.59^b^	21.73 ± 1.00^a^	21.97 ± 0.47^a^	26.47 **
Stomatal conductance (gs, mmol m^−2^·s^−1^)	600.00 ± 14.42^c^	731.33 ± 36.69^b^	814.67 ± 52.44^a^	24.49 **
N content	3.19 ± 0.10^c^	4.55 ± 0.08^b^	5.29 ± 0.03^a^	574.51 ***
C content	37.50 ± 0.30^b^	41.05 ± 0.52^a^	41.46 ± 0.70^a^	50.36 ***
Carbon/Nitrogen (C/N)	11.76 ± 0.35^a^	9.01 ± 0.10^b^	7.84 ± 0.09^c^	261.47 ***
Soluble sugar (mg·mg^−1^)	0.10 ± 0.01^b^	0.10 ± 0.01^b^	0.13 ± 0.01^a^	25.4 **
Free amino acids (μmol·mg^−1^)	3.56 ± 0.05^b^	4.07 ± 0.15^a^	3.51 ± 0.30^b^	7.35 *
Total protein (μg·mg^−1^)	1.78 ± 0.02^b^	1.83 ± 0.10^b^	2.14 ± 0.08^a^	19.29 **
Nitrogen use efficiency (NUE, g·g^−1^)	47.45 ± 2.13^a^	32.34 ± 1.11^b^	29.23 ± 1.02^c^	125.14 ***
Photosynthetic nitrogen use efficiency (PUNE, μmol g^−1^·s^−1^)	10.73 ± 0.51^a^	9.0 ± 0.30^b^	7.83 ± 0.19^c^	49.27 ***

**Table 2:** Transcription factors (TFs) differentially expressed under low N and high N.

TF Family	Low N		High N	
	Up	Down	Up	Down
bHLH	6	1	1	1
bZIP	1	1	0	2
C2H2	4	0	0	0
CO-like	1	0	1	0
DBB	1	1	0	0
E2F/DP	0	1	1	0
EIL	0	0	0	1
ERF	4	1	1	0
G2-like	0	1	1	0
GRAS	1	0	1	0
HD-ZIP	1	1	0	0
HSF	1	0	0	0
LSD	1	0	0	0
M-type_MADS	0	0	1	0
MYB	1	0	0	0
MYB_related	3	1	0	6
NAC	5	1	0	3
NF-YA	1	0	0	0
NF-YC	0	1	1	0
Nin-like	0	0	1	1
Whirly	0	1	0	0
WRKY	6	0	0	0
Total	38	10	8	15

## Supplementary Materials

Supplementary materials can be found at https://www.mdpi.com/1422-0067/20/9/2349/s1.
