# Supplementary material for: An Integrated Analysis of the Rice Transcriptome and Metabolome Reveals Differential Regulation of Carbon and Nitrogen Metabolism in Response to Nitrogen Availability
Source: Int J Mol Sci. 2019 May 11;20(9):2349. doi: 10.3390/ijms20092349 (PMC6539487; doi:10.3390/ijms20092349)
Supplement: Supplementary file 1 [file ijms-20-02349-s001.pdf]

Supplementary Material

An integrated analysis of the rice transcriptome and metabolome reveals the carbon and nitrogen metabolism regulation mechanism in response to nitrogen availability

Wei Xin[1], Lina Zhang[2], Wenzhong Zhang[1*], Jiping Gao[1*], Jun Yi[1], Xiaoxi Zhen[1], Ziang Li[1], Ying Zhao[1], Chengcheng Peng[1], Chen Zhao[1]

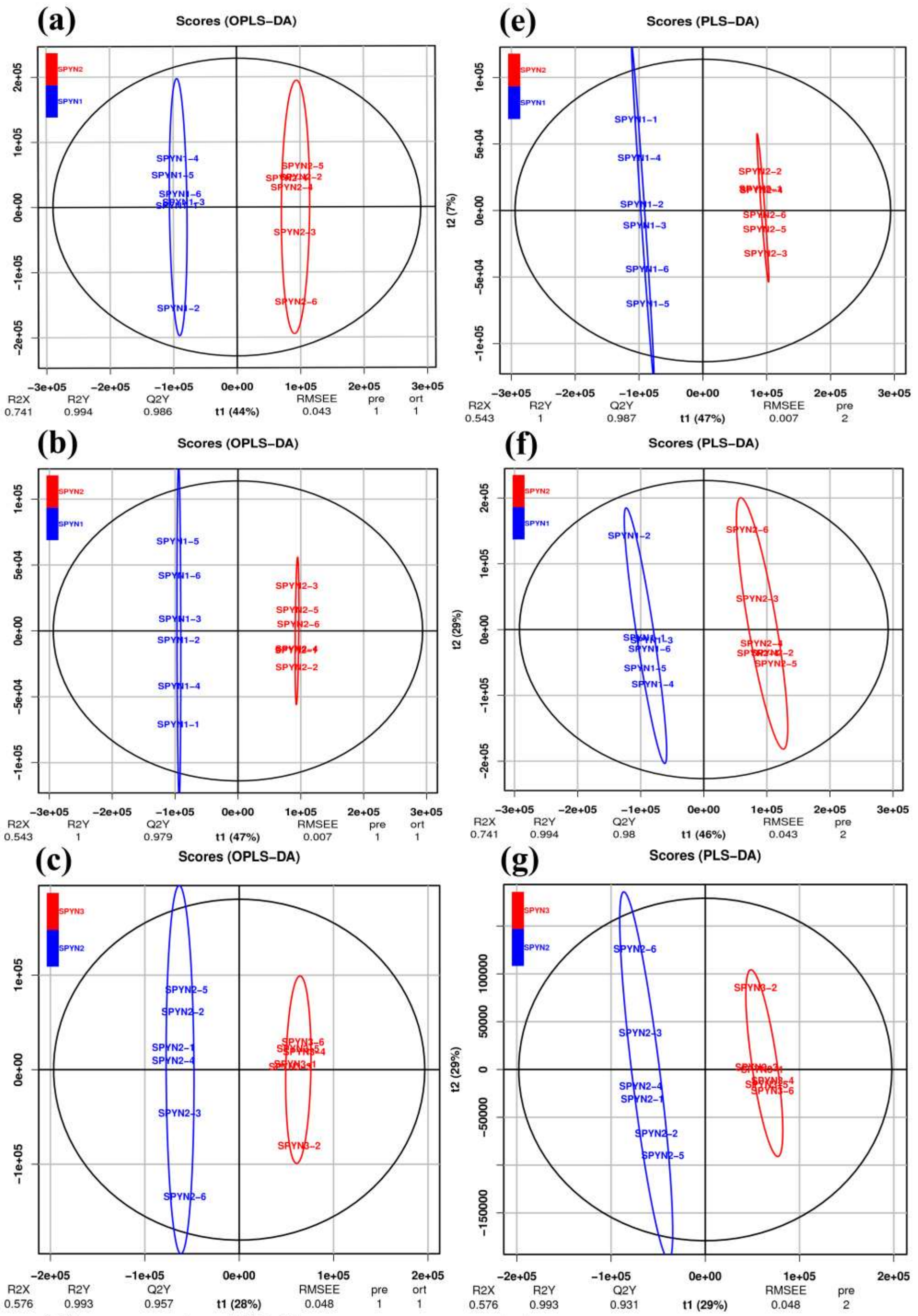

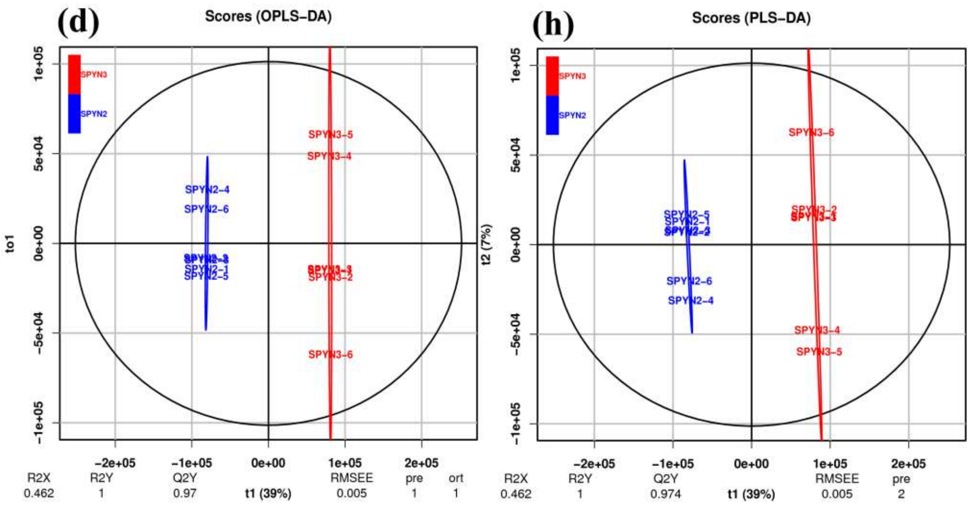

Supplementary Figure 1. OPLS-DA and PLS-DA scores plot of different nitrogen levels. The prediction parameters of the (O)PLS-DA evaluation model are R2X, R2Y and Q2; the closer the three indicators are to 1, the more stable and reliable the model, When Q2 > 0.5, the predictive ability of the model is good, and when Q2 > 0.9, the predictive ability is excellent; (a) OPLS-DA analysis of positive ion mode of control nitrogen vs. low nitrogen; blue indicates low nitrogen, and red indicates control nitrogen. (b) OPLS-DA analysis of negative ion mode of control nitrogen vs. low nitrogen; blue indicates low nitrogen, and red indicates control nitrogen. (c) OPLS-DA analysis of positive ion mode of control nitrogen vs. high nitrogen; blue indicates control nitrogen, and red indicates high nitrogen. (d) OPLS-DA analysis of negative ion mode of control nitrogen vs. low nitrogen; blue indicates control nitrogen, and red indicates high nitrogen. (e) PLS-DA analysis of positive ion mode of control nitrogen vs. low nitrogen; blue indicates low nitrogen, and red indicates control nitrogen. (f) PLS-DA analysis of negative ion mode of control nitrogen vs. low nitrogen; blue indicates low nitrogen, and red indicates control nitrogen. (g) PLS-DA analysis of positive ion mode of control nitrogen vs. high nitrogen; blue indicates control nitrogen, and red indicates high nitrogen. (h) PLS-DA analysis of negative ion mode of control nitrogen vs. low nitrogen; blue indicates control nitrogen, and red indicates high nitrogen.

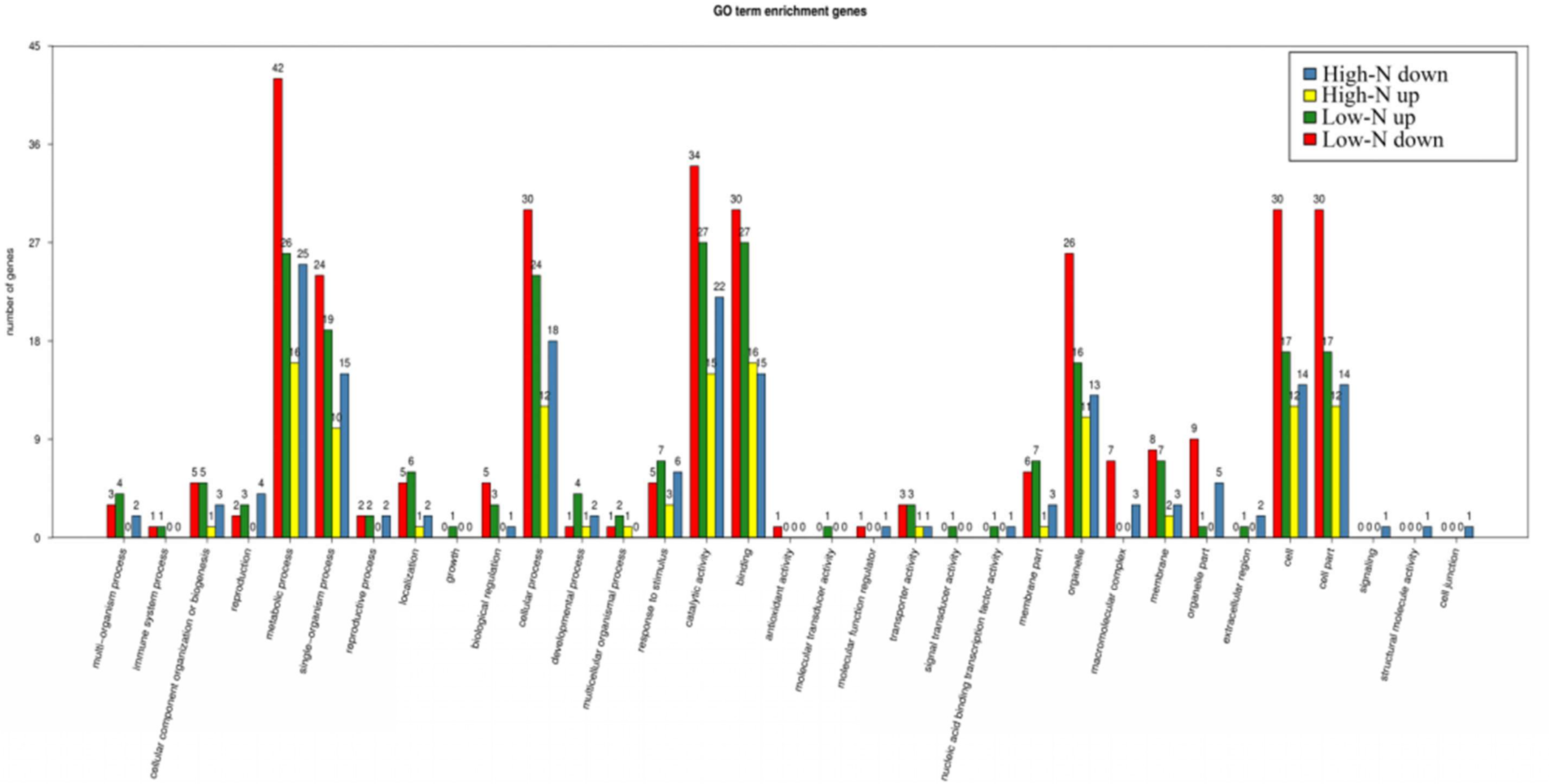

Supplementary Figure 2. Summary of GO categories gene counts of the differentially expressed genes. blue indicates down-regulated under high nitrogen, yellow indicates up-regulated under high nitrogen, red indicates down-regulated under low nitrogen, and green indicates up-regulated under low nitrogen.

Supplementary Table 1. Sequences of primers used in this study.

Gene name	Forward primer	Reverse prime
Os01g0663051	GAGGATCTCATAGTGGCGGC	CGCAACAGCAACAGTGTCAA
Os01g0820000	CTTGACCTCCTCGCAGATCC	TACAGCAACGTGTACCGCAT
Os03g0251350	CAAAACCTTGCTGCCCTTCC	CGTACTACTACCCGCCGATG
Os03g0416500	TCGTTCTTGGTCTTGGCCTC	AGAGGGTCCTGATGCTCCAT
Os03g0431200	GGCAGGGACTCAATCGAACA	GGAAGTTGCCGCTATTGCTG
Os03g0850400	ACCATTTGGGCTCACAGGAG	GTCCATGATAGCGAGGCGAA
Os04g0474800	ATGGCATGCAGTGTAAGGCT	AATGGCTATGCAACTGGCCT
Os05g0567600	GGATCTCCTCAGCGGACTTG	ACAAGATGTTCGAGGAGGCG
Os06g0270900	CAGCCAACTCAAGGACGACT	GGCTGAGCAAACTCCTCTGT
Os07g0656200	CCTTCCTGGTTCACCATCCC	CCTAGCATCTGGGACGCTTT
Os07g0684800	GTCTTTGTTGAACACCCGGC	CCTCGTCGGCATGAAGAAGA
Os11g0700500	CATGGGAGATTGCTGCATGC	CTGCATGGCAAGAGTCCTCA
OsActian1	ACCATTGGTGCTGAGCGTTT	CGCAGCTTCCATTCCTATGAA

Supplementary Table 2. Expression of TF genes under low nitrogen and high nitrogen.

**Gene_ID**	**TF_ID**	**TF_Family**	**Symbol**	**Low-N**	**High-N**
Os01g0705700	LOC_Os01g50940	bHLH	AIB	up	-
Os01g0952800	LOC_Os01g72370	bHLH	ORG2	down	-
Os02g0120500	LOC_Os02g02820	bHLH	AMS	-	down
Os03g0741100	LOC_Os03g53020	bHLH	BHLH92	up	-
Os04g0301500	LOC_Os04g23550	bHLH	BHLH35	up	-
Os09g0455300	LOC_Os09g28210	bHLH	HEC2	-	up
Os08g0490000	LOC_Os08g38210	bHLH	BIM2	up	-
Os09g0475400	LOC_Os09g29930	bHLH	BIM2	up	-
Os10g0575000	LOC_Os10g42430	bHLH	MYC2	up	-
Os02g0728001	LOC_Os02g49560	bZIP	BZIP43	-	down
Os03g0127500	LOC_Os03g03550	bZIP	RF2b	up	-
Os08g0176900	LOC_Os08g07970	bZIP	TGA4	-	down
Os11g0154900	LOC_Os11g05640	bZIP	-	down	-
Os03g0764100	LOC_Os03g55540	C2H2	-	up	-
Os05g0114400	LOC_Os05g02390	C2H2	ZAT12	up	-
Os05g0444200	LOC_Os05g37190	C2H2	WIP3	up	-
Os09g0431900	LOC_Os09g26210	C2H2	ZFP2	up	-
Os02g0731700	LOC_Os02g49880	CO-like	COL16	-	up
Os07g0667300	LOC_Os07g47140	CO-like	COL13	up	-
Os01g0202500	LOC_Os01g10580	DBB	BBX22	down	-
Os04g0540200	LOC_Os04g45690	DBB	BBX21	up	-
Os02g0739700	LOC_Os02g50630	E2F/DP	E2FE	-	up
Os06g0245900	LOC_Os06g13670	E2F/DP	E2FE	down	-
Os08g0508700	LOC_Os08g39830	EIL	EIL3	-	down
Os02g0654700	LOC_Os02g43790	ERF	ERF1A	up	-
Os02g0656600	LOC_Os02g43940	ERF	ERF034	up	-
Os02g0677300	LOC_Os02g45450	ERF	DREB1G	-	up
Os03g0182800	LOC_Os03g08460	ERF	ERF073	up	-

Os09g0287000	LOC_Os09g11480	ERF	ERF073	down	-
Os09g0522200	LOC_Os09g35030	ERF	DREB1A	up	-
Os02g0325600	LOC_Os02g22020	G2-like	ARR1	down	up
Os05g0500600	LOC_Os05g42130	GRAS	SCL32	-	up
Os07g0589200	LOC_Os07g40020	GRAS	SCL32	up	-
Os02g0149900	LOC_Os02g05640	HD-ZIP	HOX18	down	-
Os02g0649300	LOC_Os02g43330	HD-ZIP	HOX24	up	-
Os02g0232000	LOC_Os02g13800	HSF	HSFC2A	up	-
Os08g0159500	LOC_Os08g06280	LSD	LSD1	up	-
Os08g0494100	LOC_Os08g38590	M-type_MADS	MADS21	-	up
Os05g0140100	LOC_Os05g04820	MYB	MYB86	up	-
Os01g0663051	LOC_Os01g47370	MYB_related	RL3	up	down
Os01g0975300	LOC_Os01g74410	MYB_related	MYB59	-	down
Os02g0685200	LOC_Os02g46030	MYB_related	RVE1	up	-
Os05g0567600	LOC_Os05g49240	MYB_related	RL3	up	down
Os05g0579700	LOC_Os05g50350	MYB_related	RL6	-	down
Os11g0700500	LOC_Os11g47460	MYB_related	MYBAS1	down	down
Os12g0567300	LOC_Os12g37970	MYB_related	MYBAS2	-	down
Os01g0675800	LOC_Os01g48446	NAC	NAC68	up	-
Os01g0884300	LOC_Os01g66120	NAC	NAC48	up	-
Os03g0109000	LOC_Os03g01870	NAC	NAC021	-	down
Os03g0327800	LOC_Os03g21060	NAC	ONAC010	-	down
Os06g0675600	LOC_Os06g46270	NAC	NAC021	up	-
Os07g0225300	LOC_Os07g12340	NAC	NAC67	up	-
Os07g0684800	LOC_Os07g48550	NAC	NAC100	up	down
Os08g0436700	LOC_Os08g33910	NAC	FEZ	down	-
Os12g0618600	LOC_Os12g42400	NF-YA	NFYA10	up	-
Os03g0251350	LOC_Os03g14669	NF-YC	NFYC4	down	up
Os02g0136000	LOC_Os02g04340	Nin-like	NLP6	-	up
Os09g0549450	LOC_Os09g37710	Nin-like	NLP1	-	down
Os06g0145800	LOC_Os06g05350	Whirly	WHY1	down	-
Os01g0821600	LOC_Os01g60640	WRKY	WRKY46	up	-
Os01g0826400	LOC_Os01g61080	WRKY	WRKY33	up	-
Os08g0386200	LOC_Os08g29660	WRKY	WRKY53	up	-
Os09g0417600	LOC_Os09g25060	WRKY	WRKY40	up	-
Os11g0117400	LOC_Os11g02520	WRKY	WRKY46	up	-
Os11g0117500	LOC_Os11g02530	WRKY	WRKY70	up	-
